# Items

**Published:** 1899-07

**Authors:** 


### ITEMS.

Messrs. Stoddart Brothers, of Buffalo, the well-known druggists of 84 Seneca Street, suffered great loss by fire and water Saturday night, June 10, 1899. Their store, however, was promptly opened for business Monday morning, June 12th, under an agreement with the underwriters who began at once appraising the damage which, though great, is believed to be covered by the ample insurance which they carry. The promptitude of this action reflects great credit upon all parties concerned, and especially upon the Messrs. Stoddart, whose business enterprise in their branch is unexceled.

Scott's Emulsion Vindicated.—The medical profession and the trade have for the past year and a half been much interested in the controversy between Messrs. Scott & Bowne, manufacturers of Scott's emulsion, and the state dairy and food commissioner of Ohio. The trouble arose from the charges made by the Ohio food commissioner that Scott's emulsion contained a narcotic, which, if true, made it a misdemeanor under the laws of Ohio to offer it for sale without the regulation poison label. Messrs. Scott & Bowne, feeling it a duty which they owed not only to themselves, but to the profession in general, repudiated the charges in every instance, and since then the matter has been a subject for the courts to decide.

The suit brought by the commissioner against a druggist of Cincinnati for selling Scott's emulsion, which the commissioner claimed contained morphine, was lately settled in the courts at Cincinnati by a verdict for the defendants, entirely vindicating them and showing the injustice of these injurious attacks upon Scott's emulsion, the jury being out but a very few moments. The testimony brought out at the trial was overwhelmingly in favor of the claims of the manufacturers, that Scott's emulsion had never contained a narcotic of any kind. More than a score of the best chemists in the country certified to these facts. Messrs. Scott & Bowne deserves congratulations on their victory.

